# Artificial Intelligence-Powered Imaging Biomarker Based on Mammography for Breast Cancer Risk Prediction

**DOI:** 10.3390/diagnostics14121212

**Published:** 2024-06-07

**Authors:** Eun Kyung Park, Hyeonsoo Lee, Minjeong Kim, Taesoo Kim, Junha Kim, Ki Hwan Kim, Thijs Kooi, Yoosoo Chang, Seungho Ryu

**Affiliations:** 1 Department of Radiology, We Comfortable Clinic, Seoul 07327, Republic of Korea; 2 Lunit Inc., Seoul 06241, Republic of Korea; hslee@lunit.io (H.L.); mjkim0918@lunit.io (M.K.); taesoo.kim@lunit.io (T.K.); junha.kim@lunit.io (J.K.); khkim@lunit.io (K.H.K.); tkooi@lunit.io (T.K.); 3 Center of Cohort Studies, Kangbuk Samsung Hospital, Sungkyunkwan University School of Medicine, Seoul 04514, Republic of Korea; yoosoo.chang@gmail.com (Y.C.); sh703.yoo@gmail.com (S.R.); 4 Department of Occupational and Environmental Medicine, Kangbuk Samsung Hospital, Sungkyunkwan University School of Medicine, Seoul 03181, Republic of Korea; 5 Department of Clinical Research Design & Evaluation, Samsung Advanced Institute for Health Science & Technology (SAIHST), Sungkyunkwan University, Seoul 06355, Republic of Korea

**Keywords:** artificial intelligence, risk prediction, breast cancer, mammography

## Abstract

The purposes of this study were to develop an artificial intelligence (AI) model for future breast cancer risk prediction based on mammographic images, investigate the feasibility of the AI model, and compare the AI model, clinical statistical risk models, and Mirai, a state of-the art deep learning algorithm based on screening mammograms for 1–5-year breast cancer risk prediction. We trained and developed a deep learning model using a total of 36,995 serial mammographic examinations from 21,438 women (cancer-enriched mammograms, 17.5%). To determine the feasibility of the AI prediction model, mammograms and detailed clinical information were collected. C-indices and area under the receiver operating characteristic curves (AUCs) for 1–5-year outcomes were obtained. We compared the AUCs of our AI prediction model, Mirai, and clinical statistical risk models, including the Tyrer–Cuzick (TC) model and Gail model, using DeLong’s test. A total of 16,894 mammograms were independently collected for external validation, of which 4002 were followed by a cancer diagnosis within 5 years. Our AI prediction model obtained a C-index of 0.76, with AUCs of 0.90, 0.84, 0.81, 0.78, and 0.81, to predict the 1–5-year risks. Our AI prediction model showed significantly higher AUCs than those of the TC model (AUC: 0.57; *p* < 0.001) and Gail model (AUC: 0.52; *p* < 0.001), and achieved similar performance to Mirai. The deep learning AI model using mammograms and AI-powered imaging biomarkers has substantial potential to advance accurate breast cancer risk prediction.

## 1. Introduction

Breast cancer screening with mammography has demonstrated clear evidence of a reduction in breast cancer mortality with randomized trials and screening cohort studies [1,2,3]. In spite of its principle role in screening, the sensitivity of digital mammography is limited in women with dense breasts [4]. Early detection enables the diagnosis of smaller-sized tumors, with fewer nodal metastases and lower histologic grades, thereby enhancing the effectiveness of treatment [5,6,7]. At present, guidelines have been proposed by the Breast Cancer Surveillance Consortium (BCSC) and the U.S. Preventive Service Task Force (USPSTF) for risk stratification for developing breast cancer to identify high-risk groups and determine the eligibility for supplemental screening and chemoprevention [8]. 

However, the current standard statistical models have some limitations. These models are based on clinical risk factors, including reproductive factors, a family history of breast cancer, previous benign breast disease, and genetic determinants, but these factors are not routinely available in the screening workflow [9,10]. On the basis of these factors, a 5-year, 10-year, or lifetime risk of developing breast cancer is typically generated, but the discriminatory performances of these models are often reported below an area under the curve (AUC) of 0.7 [10]. 

Prior work has shown that AI algorithms are trained for not only longer time horizons to predict future breast cancer but also computer-aided detection or diagnosis (AI-CAD), and those that have been trained for shorter time horizons had a better AUC performance than those of clinical statistical models, such as BCSC [11]. In our initial experiments, we developed an AI algorithm that was trained to recognize both affected breasts and contralateral unaffected breasts as positive, while the bilateral breasts of non-cancer patients were defined as negative [12]. There have been several efforts to identify the mammographic parenchymal features associated with breast cancer [13,14]. These studies have demonstrated mammographic parenchymal extracted texture features in breast cancer patients. As AI evolves, it might detect enhanced mammographic features beyond the breast density. Recent studies have revealed that deep learning artificial intelligence (AI) models have better performance compared with traditional risk models [15,16,17,18]. 

In this study, we developed and validated a deep learning AI algorithm trained on serial mammography examinations for future breast cancer prediction as an AI powered-imaging biomarker (AI-IBM). The cutting-edge AI algorithm developed by Mirai [15] is a deep learning neural network trained on over 210,000 screening mammograms with 5379 cancer cases (cancer incidence, 2.6%), whereas, in this study, we used a cancer-enriched dataset for the purpose of effective training based on distinctive mammographic parenchymal patterns from both screening and diagnostic mammograms. We aimed to investigate our AI model’s discriminative performance and to compare it with those of clinical statistical models and Mirai.

## 2. Materials and Methods

### 2.1. Study Cohort

To construct an in-house dataset to evaluate the models, mammographic examinations were retrospectively collected from the United States between January 2010 and December 2021. To calculate the clinical statistical risk models, we collected the dataset with detailed clinical information related to breast cancer. Mammographic examinations and the clinical dataset were de-identified and collected according to the Health Insurance Portability and Accountability Act Safe Harbor standard; therefore, there was no Protected Health Information (PHI), which requires institutional review board (IRB) approval. The study cases were collected separately from the development dataset (described in the AI Model Development Section) for independent external validation, and none of the study cases were used for model development. We collected cases with the following criteria: cancer-positive cases defined with pathologic confirmation only, cancer-negative cases included benign cases that have pathologic confirmation or at least 1 year of follow-up, and normal cases that have at least 1 year of follow-up. Studies were eligible for inclusion if they met the following criteria: (a) female sex; (b) any ethnic origin; (c) 22 years old or older; (d) no personal history of breast cancer; (e) four-view (right craniocaudal, right mediolateral oblique, left craniocaudal, and left mediolateral oblique) screening images or diagnostic mammography examinations with full-field digital mammography images. For the cancer group, we only included cases with at least a 6-month time period from the cancer diagnosis to evaluate the AI future breast cancer prediction performance through the negative examinations. A total of 16,894 mammograms from 6864 women were included, of which 4002 were followed by a cancer diagnosis within 5 years.

### 2.2. AI Model Development

**Figure 1:**
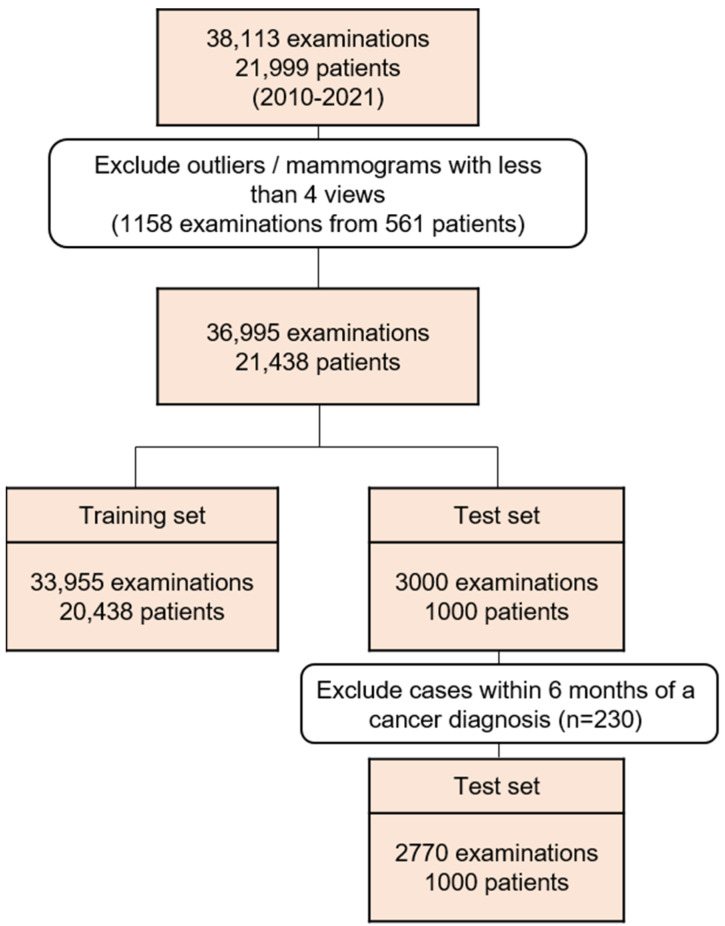
Flowchart of development dataset construction.

We developed a deep-learning AI algorithm that was trained to discriminate mammographic patterns from prior images of breast cancer patients to predict future breast cancer over time. The AI model is based on a deep convolutional neural network with an ImageNet pretrained ResNet-34 as the image feature backbone. The backbone network extracts features from the mammograms, and the fully connected layer produces the final feature vector. Two additional fully connected layers were applied to calculate the base hazard and time-dependent hazard. The predicted cumulative hazard was obtained by adding the base hazard and time-dependent hazard to a final feature vector. To train the model, 38,113 serial mammographic examinations from 21,999 women who had had at least one prior mammogram from January 2010 to December 2021 in the United States were used. We excluded 1158 examinations from 561 patients because they had outliers or less than four mammographic image views. As a result, 36,995 examinations from 21,438 women were included (Figure 1). Full-field digital mammographic examinations were performed using Hologic (Marlborough, MA, USA) (27,258 of 36,995, 73.7%) units and Siemens (Munich, Germany) (9697 of 36,995, 26.2%) units. We collected both screening and diagnostic mammograms. To accurately determine the ground-truth labels, we collected examinations with the following criteria: cancer-positive examinations defined with pathologic confirmation only, cancer-negative examinations including benign ones that have pathologic confirmation or at least 1 year of follow-up, and normal ones that have been defined with at least 1 year of follow-up. The entire development dataset was divided into a training set (33,995 of 36,995, 91.9%), with 5711 (16.8% of 33,995 training dataset) cancer cases for training the AI model, and a test set (3000 of 36,995, 8.1%), with 750 (25% of 3000 test dataset) cancer cases for the final model evaluation. We trained the model with serial mammographic images, ground-truth breast cancer information, and the diagnosis and prior examination time intervals. More details on the network architecture can be found in the study by Lee et al. [19].

### 2.3. Risk Prediction Models Operation

Our AI model accepts as inputs the four standard mammography views, left and right mediolateral oblique and left and right craniocaudal, and shows the AI score of 0–1, which indicates the probability of breast cancer risk within 1 year to 5 years. Exam-level and image-level AI scores were generated from the algorithm. To discover the performance of the AI model, exam-level AI scores were used in this study.

The Tyrer–Cuzick (TC) model (version 8) [20], the Gail model [21], and Mirai [15] were used as the comparisons for our AI model. Mirai, an academic AI algorithm freely available for research, generates AI scores to predict future breast cancer risk for up to 5 years. Since Mirai was trained and tested using mammograms of Hologic units, Mirai was validated on the mammograms using Hologic units in our external validation dataset. Time-grid sampling based on Kaplan–Meier [22] was applied to our AI model to encourage the model to make an accurate prediction; however, to compare the risk prediction models with our AI model, we used our baseline AI model without time-grid sampling to apply identical information to the other models for one sample at a time.

### 2.4. Statistical Analysis

To evaluate our AI prediction model, time-dependent AUCs for 1–5-year outcomes were evaluated in the independent external validation dataset. To compute the n-year AUC, we considered a mammogram result to be positive if it was followed by a cancer diagnosis within n years, and we considered a mammogram to be negative if it had at least n years of follow-up without a cancer diagnosis. Uno’s concordance index (C-index), which generalizes AUCs across all time points, was also evaluated.

To compare the performances of the clinical statistical risk models, the time-dependent AUCs for 1–5-year risk prediction of for TC model and 5-year AUC for the Gail model were computed. The 95% confidence intervals (CIs) and *p* values for the AUCs were calculated by using DeLong’s test [23]. The software R (version 4.2.2; R Core Team) was used by a biostatistician (M.J.K) to perform statistical analyses. A *p* value of less than 0.05 was deemed statistically significant.

## 3. Results

### 3.1. Cohort Characteristics

A total of 16,894 mammograms from 6864 women (mean age: 56 years ± 13.8 [SD]) were included for the external validation. A total of 4002 of 16,894 (23.7%) examinations were followed by a cancer diagnosis within 5 years. Of 16,894 mammograms, 6864 (40.6%) were defined as index examinations in this study, which refer to mammogram examinations at the time of diagnosis. The other 10,030 of 16,894 (59.4%) examinations were prior examinations within 5 years of the time of diagnosis. In the cancer group, 1718 of 4002 (42.9%) mammograms were index examinations, and 2284 of 4002 (57.1%) were examinations prior to diagnosis. All of the examinations in this study cohort were achieved via Hologic units (Table 1).

### 3.2. AI Algorithm Model Evaluation

In the test set of development dataset, our AI prediction model showed a C-index of 0.68 (95% CI: 0.66, 0.70). The AI model demonstrated time-dependent AUCs of 0.87 (95% CI: 0.82, 0.92) for the 1-year risk, 0.83 (95% CI: 0.79, 0.87) for the 2-year risk, 0.72 (95% CI: 0.69, 0.75) for the 3-year risk, 0.73 (95% CI: 0.70, 0.75) for the 4-year risk, and 0.81 (95% CI: 0.79, 0.82) for the 5-year risk.

In the external validation dataset, the AI model achieved AUCs of 0.90 (95% CI: 0.88, 0.91) for 1-year risk prediction, 0.84 (95% CI: 0.82, 0.85) for 2-year risk prediction, 0.81 (95% CI: 0.79, 0.82) for 3-year risk prediction, 0.78 (95% CI: 0.77, 0.80) for 4-year risk prediction, and 0.81 (95% CI: 0.79, 0.82) for 5-year risk prediction. The C-index of the AI prediction risk model with positive–negative examinations was 0.76 (95% CI: 0.74, 0.78) (Table 2).

### 3.3. AI Algorithm and Clinical Risk Model Performances

For 5-year prediction, the TC model demonstrated an AUC of 0.57 (95% CI: 0.54, 0.60), and the Gail model showed an AUC of 0.57 (95% CI: 0.54, 0.60). Our AI prediction risk model showed significantly higher 5-year AUCs than those of the TC model (*p* < 0.001) and the Gail model (*p* < 0.001). The TC model showed 0.46 (95% CI: 0.30, 0.61) for 1-year risk prediction, 0.50 (95% CI: 0.44, 0.56) for the 2-year risk, 0.48 (95% CI: 0.44, 0.51) for the 3-year risk, and 0.53 (95% CI: 0.50, 0.56) for the 4-year risk. All of the time-dependent AUCs of our AI prediction model showed significant higher performance than those of the TC model (*p* < 0.001). Our AI prediction model showed a C-index of 0.75 (95% CI: 0.74, 0.76). The AI model achieved an AUC of 0.90 (95% CI: 0.88, 0.91) for 1-year risk prediction, 0.84 (95% CI: 0.82, 0.86) for the 2-year risk, 0.81 (95% CI: 0.79, 0.82) for the 3-year risk, 0.78 (95% CI: 0.77, 0.80) for the 4-year risk, and 0.81 (95% CI: 0.79, 0.83) for the 5-year risk. Mirai demonstrated a C-index of 0.74 (95% CI: 0.73, 0.75) and an AUC of 0.90 (95% CI: 0.88, 0.92) for 1-year risk prediction, 0.82 (95% CI: 0.81, 0.84) for the 2-year risk, 0.78 (95% CI: 0.77, 0.80) for the 3-year risk, 0.77 (95% CI: 0.75, 0.78) for the 4-year risk, and 0.80 (95% CI: 0.78, 0.82) for the 5-year risk. The AUC of the 3-year prediction of our AI model is significant higher than that of Mirai (*p* = 0.019). The other time-dependent AUCs of our AI model were higher than those Mirai without statistical significance. The results are reported in Table 3.

## 4. Discussion

In this study, we developed an AI model based on mammography for future breast cancer risk prediction and investigated its performance in the independent dataset derived from the development dataset. The AI model showed a continuous, strong predictive performance at 1–5 years (AUC range: 0.78–0.90). The AI discriminative performance, as measured according to the 5-year AUCs, was higher than those of the other clinical risk models, including the TC model and the Gail model. Mirai has showed promising results in short-term breast cancer risk prediction within 5 years, and the AI model developed using the cancer-enriched dataset used in this study demonstrated similar or better performance. 

**Figure 2:**
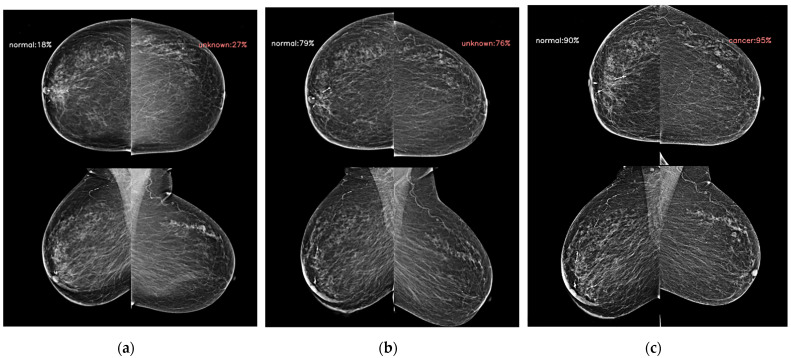
The AI model results for serial digital mammography images with AI scores to predict future breast cancer in a 62-year-old woman with subsequent left breast cancer. (**a**) The AI score of left breast presented as 0.27 4 years ago following the cancer diagnosis. (**b**) The score increased to 0.76 2 years ago. (**c**) The score achieved 50 days ago from the cancer diagnosis was 0.95. Note that the contralateral breast scores were 0.18, 0.79, and 0.90, respectively.

**Figure 3:**
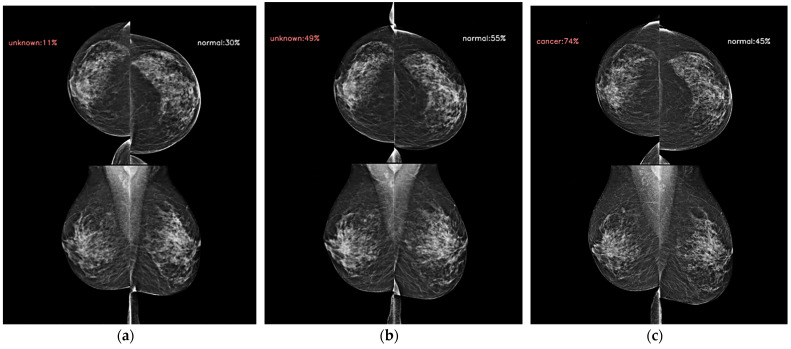
AI model results for serial digital mammography images with AI scores to predict future breast cancer in a 53-year-old woman with subsequent right breast cancer. (**a**) AI score of right breast presented as 0.11 4 years ago following the cancer diagnosis. (**b**) The score increased to 0.49 2 years ago, and (**c**) 0.74 28 days ago following the cancer diagnosis. The contralateral breast showed scores of 0.30, 0.55, and 0.45, respectively. Note that mammographic densities did not differ or minimally decreased at the serial examinations, and mammographic parenchymal patterns were difficult to discriminate from each other with human eyes; however, AI scores increased over the time not only in the affected breast but also in the contralateral breast.

**Figure 4:**
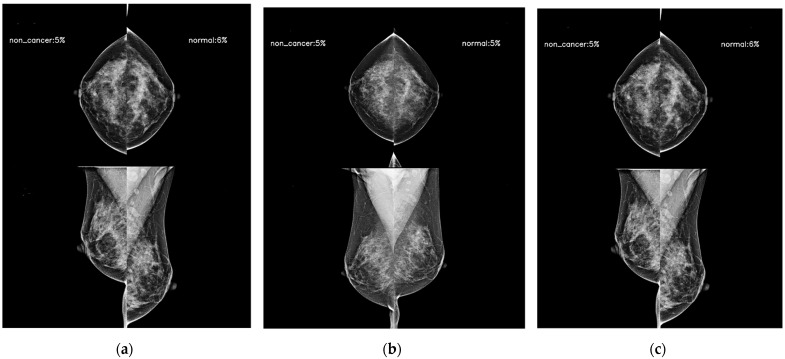
AI model results for serial digital mammography images with AI scores to predict future breast cancer in a 47-year-old woman with a pathologically proven benign lesion in her right breast. (**a**) AI score of right breast presented as 0.05 4 years ago following the diagnosis. (**b**) The score was 0.05 2 years ago, and (**c**) the score achieved at the time of biopsy was 0.10. Note the contralateral breast showed scores of 0.06, 0.05, and 0.09, respectively. This case showed extremely dense breasts on serial mammograms; however, the AI scores from three the examinations remained below 0.10 continuously.

**Figure 5:**
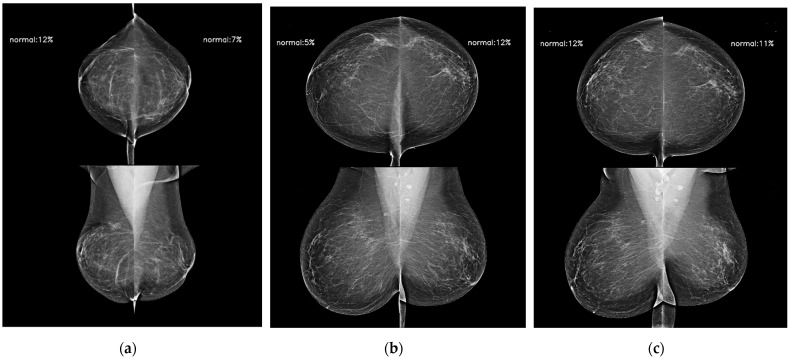
AI model results for serial digital mammography images with AI scores used to predict future breast cancer in a 65-year-old woman with negative results during 5 years of follow-up. AI scores of bilateral breasts (**a**) 4 years ago, (**b**) 2 years ago, and (**c**) at the time of diagnosis were kept up to 0.12 all of the time.

Mammographic density is one of the strongest risk factors [24], and its addition has improved the traditional breast cancer risk models. A beneficial effect from the supplemental screening of women with dense breasts has also been reported [25]. Beyond the mammographic breast density, the mammographic parenchymal pattern has been investigated for risk assessment. Dembrower et al. [26] showed that a deep learning risk score based on mammographic images, the age at examination, and future breast cancer acquisition parameters had an AUC (0.65) higher than that of the mammographic density score (0.60). Unlike AI-CAD for breast cancer detection and diagnosis, which is trained using a dataset with the cancer ground truth and the cancer location via exam-level, image-level, and pixel-level labels [27], the AI prediction model in this study was trained via exam-level labels so that it could learn the overall mammographic parenchymal patterns and identify the mammographic features that predict future cancer development. Figure 2 and Figure 3 showed representative cases of subsequent breast cancer, notably on AI scores of contralateral breast to affected breast. These cases showed a gradual increase in score of bilateral breast. It suggests that the AI risk prediction model could identify high-risk women for breast cancer not via the early detection of cancer but by discriminating characteristic mammographic parenchymal patterns. Figure 4 and Figure 5 were cases of women with benign and negative results, and the AI scores remained continuously low.

There have been several studies on the use of deep learning-based breast cancer risk prediction models. The current state-of-the art Mirai model [15,16] showed 1-year AUCs of 0.78–0.90 in its multi-institutional validation on a diverse cohort, and the time-dependent AUCs progressively decreased and eventually plateaued as they approached the 5-year risk prediction. The time-dependent AUC tendency of the Mirai model over time was similar to that of our AI model in this study. Predicting the 1–2-year breast cancer risk showed higher discrimination (AUC range: 0.84–0.90) than that of predicting the 3–5-year risk (AUC range: 0.78–0.81). As it is difficult to clearly distinguish between making an actual future breast cancer prediction with distinctive mammographic patterns on negative mammograms and detecting early breast cancer findings, it might be easier for AI to identify mammographic features for shorter time periods. In addition, our AI training dataset had a higher proportion of index cases at the time of diagnosis, and this influenced the time-dependent AUC tendency over time. Notwithstanding, our study showed a sufficient AI prediction model performance, with an AUC of 0.81 (95% CI: 0.79, 0.82) for the 5-year risk. Our development dataset had various mammographic examinations obtained from diverse Hologic and Siemens unit devices, whereas the Mirai model trains images from Hologic units only [11]. The C-index and time-dependent AUCs were higher in the external validation set than those in the test set, even though it was independent of the training dataset. It appears that the dataset composition (60% Hologic and 40% Siemens in the test set vs. 100% Hologic in the external validation set) had an influence on the performances. We intended to train the mammographic parenchymal pattern of women with breast cancer effectively using the model through a cancer-enriched development dataset based on our experience in the development of a robust AI model to detect and diagnose breast cancer [26]. To the best of our knowledge, there has been no study that has investigated the impacts of several clinical factors of the development dataset, including the cancer proportion, mammographic units, and examination time interval, on the performance gain of AI prediction models, and this is necessary for their robustness.

Accurate risk assessment allows for precise, personalized breast cancer screening. Clinical statistical risk prediction models have been widely used to estimate the breast cancer incidence probability and incorporated into supplemental screening guidelines [8]. Recent studies have compared deep learning AI models and clinical risk models for 5-year breast cancer risk prediction. Yala et al. achieved a significant advanced performance with a 5-year AUC of 0.76 compared with the TC model, which obtained a 5-year AUC of 0.62 [15]. In an observation study by Arasu et al., the 5-year BCSC AUC was 0.61, and that for the Mirai model was 0.67 [11]. Our study reinforces previous works that found that substantial improvements in AI prediction models based on mammography for future breast cancer prediction are needed. A simulation study by Eriksson et al. revealed that using DBT in a risk model (AUC: 0.83) for 1-year breast cancer risk prediction resulted in a risk model performance improvement based on FFDM (AUC: 0.73) [28]. Our preliminary experiments incorporating longitudinal prior images into the AI prediction risk model in this study achieved a significant performance improvement [19]. This suggests that there is potential for a further robustness gain for the mammography-based AI prediction model to predict future breast cancer through adding richer clinical and imaging features.

This study had several limitations. First, it was performed with a retrospectively collected dataset and diverse examination time intervals. In the past few years, the use of AI prediction models based on mammography in retrospective screening cohorts has been investigated in a few studies [16,18]; however, it is still unclear how to implement them in real-world screening programs instead of in the existing clinical risk models. Second, several factors that could influence the breast cancer risk, such as the breast density and breast cancer characteristics, were not taken into consideration in this study. Last, in this study, only the discrimination performances using C statistics and ROC curves were evaluated for the AI prediction model. Although our AI model showed a robust discrimination performance, calibration evaluation is necessary to evaluate the impact of the model in terms of effectively identifying women with a high risk of developing breast cancer in future works. Moreover, regarding accurate risk stratification, it is imperative that women at high risk for breast cancer based on the risk prediction model are diagnosed with breast cancers with worse prognoses.

In conclusion, the AI prediction model that we developed based on diverse and cancer-enriched mammograms showed a better discrimination performance than those of clinical risk models in future breast cancer prediction. This approach for robust risk stratification in breast cancer screening has the potential to improve personalized screening programs.

## Figures and Tables

**Table 1:** Detailed demographics for the training, test, and external validation datasets.

Characteristics	Training Set		Test Set		External Validation Set	
	All	Cancer	All	Cancer	All	Cancer
Examinations	33,955	5711	3000	750	16,894	4002
Time						
Index *	20,356 (59.9)	2652 (46.4)	1000 (33.3)	241 (32.1)	6864 (40.6)	1718 (42.9)
Prior	13,599 (40.1)	3059 (53.6)	2000 (66.7)	484 (67.9)	10,030 (59.4)	2284 (57.1)
Group						
Cancer	5686 (16.7)	5686 (100)	750 (25.0)	750 (100)	4002 (23.7)	4002 (100)
Non-cancer	28,269 (83.3)	-	2250 (75.0)	-	12,892 (76.3)	-
Age (Years)						
<40	686 (2.0)	75 (1.3)	39 (1.3)	2 (0.3)	349 (2.1)	64 (1.6)
40–50	7119 (20.9)	844 (14.9)	447 (14.9)	61 (8.1)	1624 (9.6)	308 (7.7)
50–60	8088 (23.8)	1318 (23.2)	742 (24.7)	131 (17.5)	2510 (14.9)	553 (13.8)
60–70	7576 (22.3)	1823 (32.1)	807 (26.9)	208 (27.7)	5374 (31.8)	1247 (31.2)
70–80	6686 (19.7)	1248 (21.9)	805 (2638)	271 (36.1)	5513 (32.6)	1407 (35.2)
>80	1800 (5.3)	378 (6.6)	160 (5.4)	77 (10.3)	1524 (9.0)	423 (10.5)
Manufacturer						
Hologic	25,458 (75)	5091 (89.5)	1800 (60)	450 (60)	16,894 (100)	4002 (100)
Siemens	8497 (25)	595 (10.5)	1200 (40)	300 (40)	0	0
Device						
Lorad Selenia	16,365 (48.2)	2547 (44.8)	1031 (34.4)	197 (26.3)	6479 (38.4)	922 (23.0)
Selenia Dimensions	9093 (26.7)	2544 (44.7)	769 (25.7)	253 (33.7)	10,415 (61.6)	3080 (77.0)
Mammomat Revelation	319 (0.9)	33 (0.6)	34 (1.1)	3 (0.4)	0	0
Mammomat Inspiration	7289 (21.5)	455 (8.0)	1072 (35.7)	249 (33.2)	0	0
Mammomat Novation DR	889 (2.7)	107 (1.8)	94 (3.1)	48 (6.4)	0	0

Data are the number of cases, with percentages in parentheses for case pool. * Mammograms at the time of diagnosis were defined as index examinations, and examinations prior to the time of diagnosis were also used.

**Table 2:** Model evaluation of test sets of AI and comparative time-dependent AUC performances with clinical risk models in the external validation set.

	C-Index	1-Year AUC	2-Year AUC	3-Year AUC	4-Year AUC	5-Year AUC
Test set (2770 mammograms from 1000 patients, 520 followed by cancer)						
Case pool ^1^		2220 (20)	1675 (65)	1432 (248)	1038 (342)	848 (520)
AI-IBM ^2^	0.68 (0.66, 0.70)	0.87 (0.82, 0.92)	0.83 (0.79, 0.87)	0.72 (0.69, 0.75)	0.73 (0.70, 0.75)	0.81 (0.79, 0.82)
External validation set (16,894 mammograms from 4002 followed by cancer)						
Case pool ^1^		9855 (240)	7611 (541)	5468 (969)	3508 (1337)	2508 (704)
AI-IBM ^2^	0.76 (0.74, 0.78)	0.90 (0.88, 0.91)	0.84 (0.82, 0.85)	0.81 (0.79, 0.82)	0.78 (0.77, 0.80)	0.81 (0.79, 0.82)

^1^ The number of total cases used in the test set and external validation set, followed by the number of cancer cases shown according to time points. ^2^ All other C-index and AUC metrics are followed by 95% CIs in parentheses. AUC: area under the receiver operating characteristic curve; AI-IBM: artificial intelligence-imaging biomarker.

**Table 3:** Model performance comparison for our AI model and Mirai.

	C-Index	1-Year AUC	2-Year AUC	3-Year AUC	4-Year AUC	5-Year AUC
AI-IBM ^1^ (baseline)	0.75	0.90	0.84	0.81	0.78	0.81
	(0.74, 0.76)	(0.88, 0.91)	(0.82, 0.86)	(0.79, 0.82)	(0.77, 0.80)	(0.79, 0.83)
Mirai	0.74	0.90	0.82	0.78	0.77	0.80
	(0.73, 0.75)	(0.88, 0.92)	(0.80,0.84)	(0.77, 0.80)	(0.75, 0.78)	(0.78, 0.82)
*p* value ^2^	-	0.977	0.187	0.019	0.218	0.542
Tyrer–Cuzick	0.54 (0.52, 0.56)	0.46 (0.30, 0.61)	0.50 (0.44, 0.56)	0.48 (0.44, 0.51)	0.53 (0.50, 0.56)	0.57 (0.54, 0.60)
*p* value ^2^	-	<0.001	<0.001	<0.001	<0.001	<0.001
Gail ^3^	-	-	-	-	-	0.57 (0.54, 0.60)
*p* value ^2^	-	-	-	-	-	<0.001

^1^ All other C-index and AUC metrics are followed by 95% CIs in parentheses. AUC: area under the receiver operating characteristic curve; AI-IBM: artificial intelligence-imaging biomarker. ^2^ *p* values estimated from DeLong’s test for comparison with AI. ^3^ For the Gail model, cases which were followed-up for at least 5 years were evaluated and compared with the validation of the AI model in the same cohort.

## Data Availability

The data presented in this study are available upon request.
